# Rupture of the Perineum

**Published:** 1885-07

**Authors:** Frank Hastings Hamilton


### Cullings From Contemporaries.

### RUPTURE OF THE PERINEUM.

Dr. Frank Hastings Hamilton, the eminent surgeon and gynecologist, has a paper in the New York *Medical Record* for June 27, headed "A Few Practical Remarks on Rupture of the Perineum." As everything from the pen of this distinguished authority is read with interest and profit, and as our space will not permit the reproduction of the entire paper, we have endeavored to give an outline of his views, and have extracted entire, his remarks on the "Primary Operation."

He says, it is unjust to say that rupture can always, or even generally, be avoided by the skill of the accoucheur, since its occurrence is often due to conditions and circumstances beyond his control, and enumerates them. He quotes Prof. Elliott as saying on this subject, "The perineum can always be saved from laceration when the camel can go through the eye of the needle."

Speaking of stereotyped advice to "support the perineum," Dr. Hamilton says that "support" with the hand, in the sense in which it is generally meant, is of no service in reinforcing and strengthening the perineum, and will do little, if anything, towards preventing laceration; that the idea that it does, or can, cannot be sustained on any sound mechanical theory, or by experience; if for the purpose of delaying the advance of the child, "the intention is good, but the method of seeking to accomplish it, is of no value." In that way the advance of the child could not be retarded, if the uterus is acting normally or with unusual vigor; but if the object in "supporting the perineum," with the hand, is to "carry the head forward toward the pubes" there is some sense in it, and it is especially advisable whenever there is a lack of proper curve in the sacrum (or other cause) and the vertex does not take that direction.

In the author's early days, forceps were very little used; he was a pupil of Dewees; but since that time there has been a complete revolution, and now the use of the forceps is almost universally taught and practiced; and the Doctor hints at the relation of cause and effect very largely between this and the very evident increase in the frequency of perineal lesions, but simply says that it has been "coincident."

He does not hold anæsthetics in labor as guiltless, and as safe as many other eminent modern teachers do; nor as incapable of doing harm as some of our own Texas obstetricians assert. He asserts that they are something more than anæsthetics, and that their effects are by no means limited to the relief of pain, but that "*in most cases they exert a positive influence upon the uterine contractions, and in this respect are caprecious,*" [and therefore dangerous; italics ours.—Ed.] It can be readily seen, therefore, how even anæsthetics may be, and doubtless often are, grave and potent factors in the production of laceration, as well as, on the other hand, of uterine inertia, and a consequent uterine hemorrhage, as well as delay in the expulsion of the placenta, since, the author says, "when given in small quantities, and at the beginning, they are apt to increase the frequency and strength of the contractions," while in other cases, he has seen suspension of contractions.

The following has the sound of a prophecy and a warning:

"While there can be no question that the forceps and anæsthetics have been of incalculable service to parturient women, I have a strong conviction that, since they have come into such common, and almost universal use, they have done a great deal of harm. I believe that the time is not distant in the future, when it will become apparent to all, that we have, in these matters, progressed too rapidly, and possibly, that our progress has been altogether in the wrong direction."

As to the advisability of immediate operation, Dr. Hamilton says: In the simpler form of these accidents, where only the forchette is torn, no serious injury is inflicted, and no surgical treatment is demanded. If left to itself, and some care be taken to keep the parts clean, it may be restored to its original condition by the unaided process of cicatrization; but if not thus restored, no inconvenience to the patient will issue. Indeed the partial loss of the perineum may render any further laceration in subsequent deliveries less probable."

But with regard to the graver injuries, and speaking of the operation, he says:

"I cannot agree with some modern gynecologists, that it is better in such cases to proceed at once to close the rent by sutures, and for several reasons, some of which have already been anticipated. The parts have suffered such a degree of stretching and contusion as to render the occurrence of inflammatory reaction, if not of sloughing, almost inevitable; the lochial discharges will make it impossible to keep the parts clean; the operation itself inflicts a severe injury when the condition of the patient is already critical from other causes; and finally because, under judicious management, the rent frequently becomes partially, and sufficiently closed, spontaneously."

The writer then argues that union in cases of partial laceration never occurs by first intention; "the conditions are all unfavorable to this occurrence," and closes that part of his paper as follows:

"From any point of view it comes to this, that it is proposed to make an operation, by no means trivial in its character, requiring, according to Hewson, two or three "rather deep silver-wire sutures," not to speak of the subsequent special management of the bowels required, and the removal of the sutures; and which operation, at the best, is very liable to fail; and which may subsequently, in case no operation is made, be found to have been unnecessary; or which, if it becomes necessary, can be made more thoroughly and successfully at a later period—it is proposed, I say, to make the operation under every surgical disadvantage, and when the patient, prostrated and trembling from the results of a severe labor, is in the worst possible condition, both mentally and physically, to endure the shock of an operation, or even its announcement. Nothing but the most urgent, imperative necessity can justify the operation, and this necessity has not been shown to exist."

The Doctor further adds, by way of clincher, that every accoucheur is not competent to do the operation, and experts cannot sometimes be found "within an hour"—the limit which Hewson places on the time when an operation should be done, after delivery.

We have not space for the balance of this important paper.

